# Intradural spinal varix: the “doughnut” sign on *T*_2_ weighted MR and confirmation with gadolinium-enhanced arterial and blood pool MR angiography

**DOI:** 10.1259/bjrcr.20160078

**Published:** 2016-09-22

**Authors:** Thien J Huynh, Robert A Willinsky

**Affiliations:** ^1^Department of Medical Imaging, University of Toronto, Toronto, ON, Canada; ^2^Division of Neuroradiology, Department of Medical Imaging, Toronto Western Hospital, University Health Network, University of Toronto, Toronto, ON, Canada

## Abstract

Intradural spinal varices are rare lesions, with only three cases being previously reported in the literature. Previously described patients underwent MRI for non-specific low back pain and radiculopathy and were found to have an intradural lesion adjacent to the cauda equina, mimicking a nerve sheath tumour or ependymoma. Consideration of an intradural varix in the differential diagnosis of an intradural extramedullary spinal lesion is necessary to guide appropriate management. We report a case of an intradural spinal varix diagnosed with first-pass arterial and blood pool phase gadolinium-enhanced auto-triggered elliptic centric-ordered MR angiography. Digital subtraction angiography confirmed that there was no shunt but failed to demonstrate the varix. We reviewed the existing literature to look for common clinical and imaging features.

## Clinical presentation

A 69-year-old female presented with a 9-year history of low back pain progressing over 2 years, radiating to the left hip and leg. There was no history of weakness, sensory loss, and bowel or bladder dysfunction. Past medical history was unremarkable and there was no prior surgical history. Neurological examination demonstrated normal strength and sensation in the lower extremities. Deep tendon reflexes and plantar responses were normal.

## Imaging findings and follow-up

**Figure 1.**
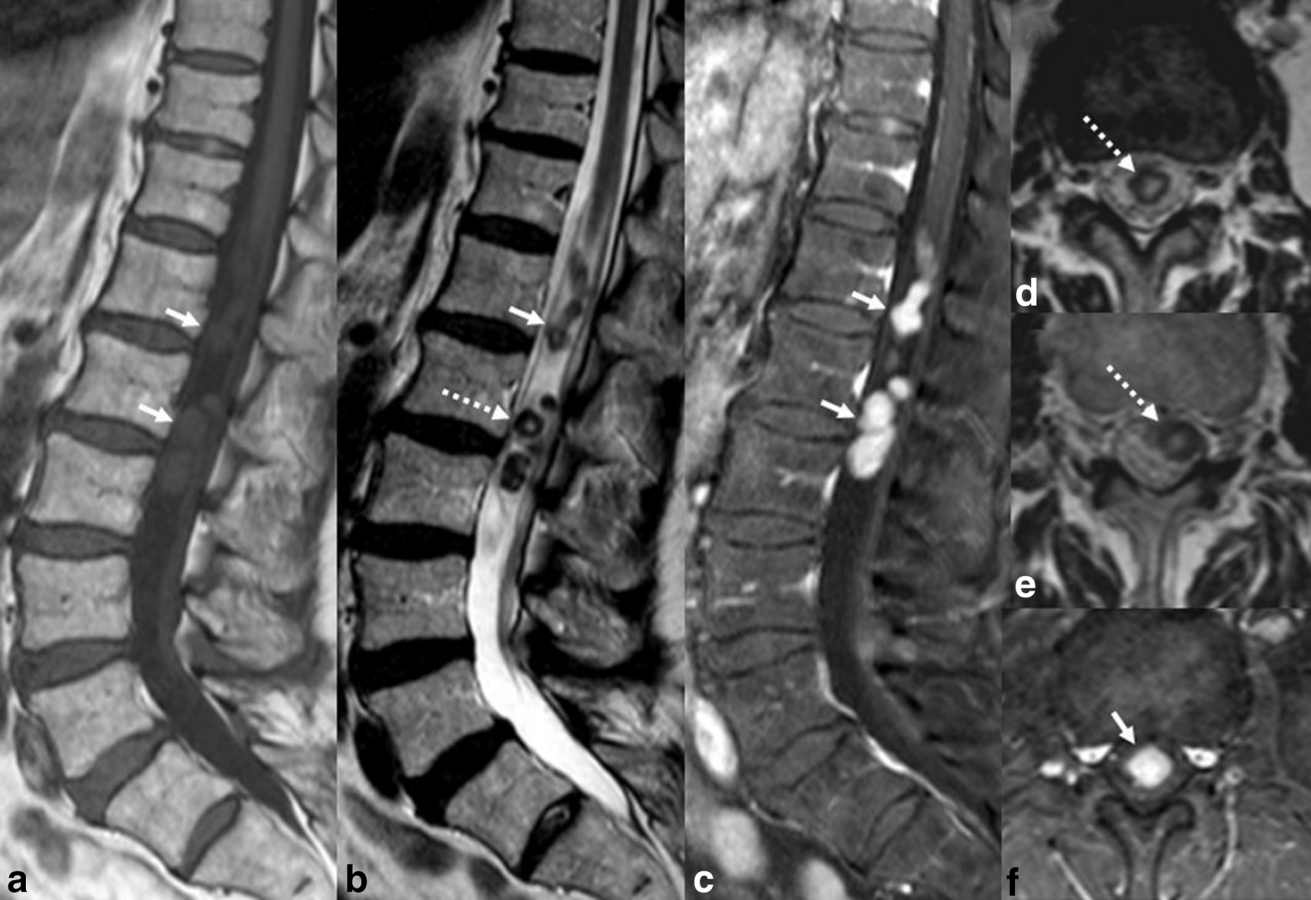
Sagittal pre-contrast *T*_1_ (a), *T*_2_ (b), and post-contrast *T*_1_ (c) images show an intradural extramedullary serpiginous tubular lesion (solid arrows) extending from T12 to L3, with intermediate signal on *T*_1_ weighted images, peripheral hypointensity and central hyperintensity on *T*_2_ weighted images (the “doughnut” sign, dotted arrow) and avid homogeneous enhancement. The “doughnut” sign is also present on multiple axial *T*_2_ slices (d and e, dotted arrows). Avid enhancement within the lesion is also visualized on axial *T*_1_ post-contrast images (f, solid arrow).

**Figure 2.**
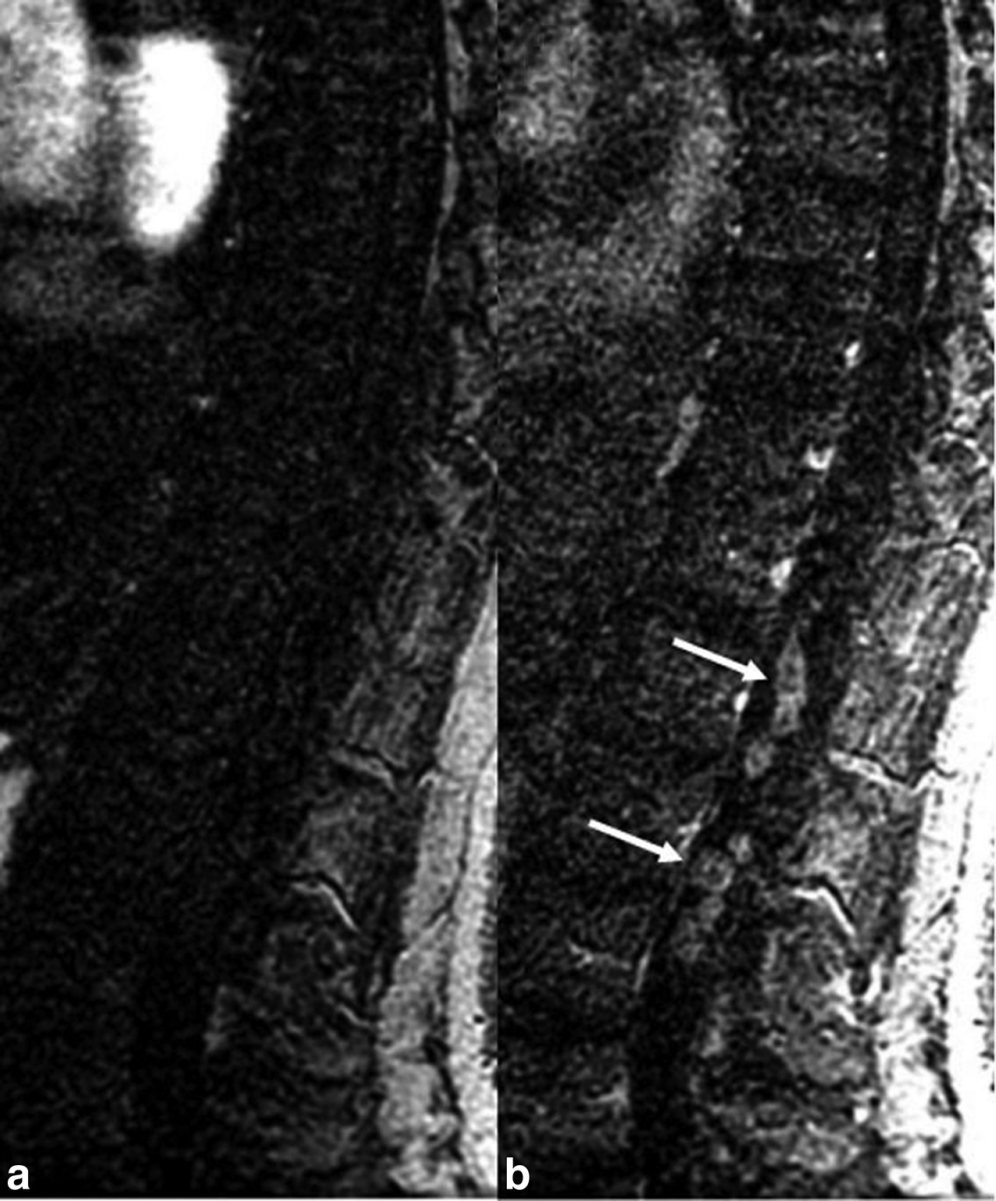
First-pass arterial phase auto-triggered elliptic centric-ordered MR angiography (a) shows no arterial shunting within the varix. Blood pool phase MR angiography (b) shows avid enhancement within the varix with areas of relatively reduced enhancement centrally (arrows), which correspond with the “doughnut” sign appearance on *T*_2_ weighted images in Figure 1.

Conventional MRI of the spine demonstrated a well-defined intradural extramedullary serpiginous lesion displacing the cauda equina nerve roots peripherally and extending from T12 to L3 (Figure 1). The lesion was isointense to the spinal cord on *T*_1_ weighted sequences and had peripheral hypointensity with central hyperintensity on *T*_2_ weighted sequences. The lesion enhanced intensely on delayed post-contrast *T*_1 _weighted images. On axial images, the lesion was serpiginous and extended from the T12 to L3 level. There was no spinal cord oedema and no perimedullary flow voids. A 6-month follow-up MRI with arterial and blood pool phase auto-triggered elliptic centric-ordered MR angiography (MRA) sequences (Figure 2) demonstrated stable appearance of the lesion on conventional sequences without evidence of shunt on MRA. On blood pool phase MRA, there was avid enhancement of the lesion with areas of relatively reduced enhancement centrally. The diffuse, progressive, delayed filling of the lesion on blood pool phase MRA and post-contrast images, its morphology and *T*_2_ weighted imaging characteristics, and stability over 6 months were diagnostic of an intradural venous varix.

**Figure 3.**
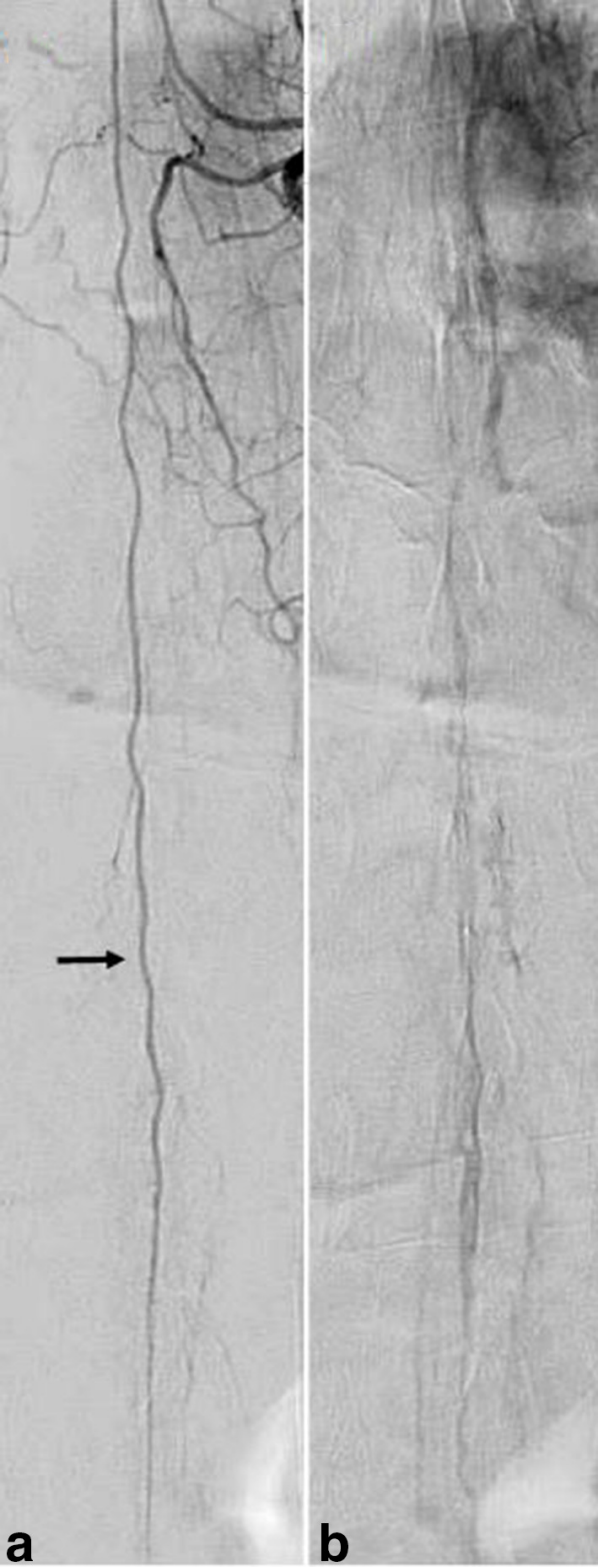
Frontal projection spinal digital subtraction angiography in the arterial phase (a) demonstrating a normal anterior spinal artery arising from the artery of Adamkiewicz, which arises from the left T8 segmental artery, without arteriovenous shunting. The spinal varix is not visualized during the venous phase (b).

Conventional digital subtraction angiography (DSA) definitively excluded an arteriovenous shunt (Figure 3). The DSA included a lumbar aortogram, selective angiograms of the thoracic intercostal and lumbar arteries from T6 to L4 and bilateral internal iliac artery angiograms. The DSA showed normal arteries and veins without evidence of filling of the intradural varix.

## Discussion and follow-up

We report a case of a spinal intradural venous varix characterized with first-pass arterial and blood pool phase MRA in a patient with longstanding low back pain and radiculopathy. Spinal intradural varices are rare lesions with only three cases being previously reported (Table 1).^1–3^ They must be differentiated from intradural tumours or spinal arteriovenous fistulas (AVFs). The differential diagnoses for intradural tumours include nerve sheath tumours, haemangioblastomas, ependymomas, meningiomas, paragangliomas, lymphoma and drop metastases.^1^ Spinal AVFs could include both dural and spinal cord AVFs. In children, perimedullary AVFs often have high flow with large feeding arteries and marked dilatation of the veins.^4^ Auto-triggered elliptic centric-ordered MRA and DSA in our case confirmed the lack of arterial–venous shunting.^5^ Avid enhancement of the lesion on blood pool phase MRA and post-contrast sequences, in addition to the elongated serpiginous morphology with intermediate signal on *T*_1_ weighted imaging, peripheral hypointensity and central hyperintensity on *T*_2_ weighted imaging were characteristic of an intradural venous varix described by Moonis et al,^1^ which was previously confirmed by surgical intradural exploration. A similar pattern of enhancement on delayed post-contrast imaging is encountered in varices found in more common locations such as the orbit.^6^ The DSA failed to demonstrate the large varix owing to its poor contrast compared to the excellent contrast achieved by MRA.

**Table 1.** Review of prior intradural venous varix case reports

Case report	Diagnosis	Age/g ender	Presentation	MRI	Treatment
Moonis et al 2003^1^	Non-thrombosed intradural varix	87/M	2 years of low back pain, worsening right leg pain over 2 months in the L5 distribution and increased pain with bending, sitting and straight-leg raising	Serpentine intradural lesion at L3–L4, isointense *T*_1_ signal to cord, central high/peripheral low *T*_2_ signal, intense homogeneous enhancement. No change after surgery at 4 months	Surgery—L3–5 laminectomy and right L5 foraminotomy—intradural exploration demonstrating enlarged intradural vein and left undisturbed. Symptoms resolved post surgery. Stable imaging appearance of varix at 4 months
Tender 2008^2^	Thrombosed intradural varix	51/F	4-month history of severe low back pain radiating to lower extremities, frequent falls, urinary retention	Large intradural mass posterior at L3, rounded heterogeneous *T*_1_ hyperintense, heterogeneous *T*_2_ hyperintense lesion with enhancement	Surgery—“purple” lesion associated with spinal nerve entering and exiting the lesion found at surgery—en bloc resection performed. Patient back to baseline function 1-year post-operatively
Paldor et al 2010^3^	Non-thrombosed intradural varix	55/M	Low back pain radiating to buttocks and right thigh, stabbing, worse when lying, relieved by sitting or standing	Ellipsoid intradural intensely enhancing mass at L2, “grows” with Valsalva. No change after 2 years	Patient refused surgery. Symptoms resolved in 2 years with weight reduction

F, female; M, male.

Previously described intradural varices have presented with non-specific low back pain and radiculopathy.^1–3^ In the case of a thrombosed intradural venous varix, symptoms may include severe pain and urinary retention.^2^ Patient ages have ranged from 51 to 87 years, with a 1 : 1 male to female ratio after inclusion of our patient in the series. Previously reported levels of varices have spanned from L2 to L4 over one to two vertebral body segments. As demonstrated in our case, and in the case by Moonis et al,^1^ the appearance of intermediate signal on *T*_1_ weighted imaging with lack of expected flow void on conventional *T*_1_ sequences most likely relates to MR artefacts from slow venous flow.^7^ The peripheral hypointensity and central hyperintensity on *T*_2_ weighted imaging, which we have been referred to as the “doughnut” sign (Figure 1), may also be the result of a combination of flow void peripherally and slow flow centrally. This hypothesis is further supported by the pattern of enhancement on the blood pool phase MRA with central relatively reduced enhancement compared to the periphery. In our case, and the case by Moonis et al^1^, delayed post-contrast images demonstrated subsequent avid homogeneous enhancement. More focal rounded lesions described in two previous cases may present a diagnostic dilemma.^2,3^ Paldor et al^3^ described a case of an enhancing ellipsoid mass centred posterior to the vertebral body at L2 and not well seen on *T*_1_ or *T*_2_ weighted sequences. A Valsalva technique was used in this case to demonstrate growth of the lesion after breath-holding sequences to confirm the diagnosis.^3^ Tender^2^ described a rounded intradural thrombosed varix at L3 that was resected en bloc. On MR, this lesion had a heterogeneous intrinsic hyperintense signal on *T*_1_ weighted images, reflecting varying stages of clotted blood products, which were confirmed by pathology.

An intradural varix is a dilated, tortuous radicular vein. The radicular veins travel from the cord along the exiting nerve roots, which pierce the dura either with the nerve root or at separate dural foramina between the nerves. A poorly understood antireflux mechanism is present at the transdural course of the radicular vein, which is characterized by narrowing and zigzagging of the vein while crossing the dura.^8^ Beyond the dura, the radicular veins drain into a valveless extradural venous system, which is in contact with the posterior margins of the vertebral bodies and intervertebral disks, which then drains into the vena cava and azygous systems. The aetiology of an intradural varix remains uncertain; however, the adult presentation of these cases favours an acquired pathophysiology. Symptoms may result from mass effect on the adjacent nerve roots; however, in the two prior cases of non-thrombosed varices, clinical symptoms had resolved despite imaging stability of the varix at follow-up MRI at 4 months and 2 years.^1,3^

Given the uncertainty as to whether the lesion was symptomatic, the patient was managed conservatively with analgesics and has remained clinically stable at 1 year based on the latest MRI and DSA.

## Conclusions

This is the first case of an intradural venous varix evaluated with gadolinium-enhanced arterial and blood pool MRA. The *T*_2_ central hyperintensity and peripheral hypointensity (the “doughnut” sign), and serpiginous morphology are signs that can be recognized to consider this rare diagnosis.

## Learning points

1. Intradural venous varices are rare intradural vascular lesions that may mimic an intradural extramedullary tumour or a spinal AVF.
2. The *T*_2_ “doughnut sign” and evaluation with MRA with arterial and blood pool phases may be helpful in establishing the diagnosis of an intradural venous varix.

## Consent

Written informed consent was obtained from the patient for publication of this case report, including accompanying images.
